# CompCorona: A web application for comparative transcriptome analyses of coronaviruses reveals SARS-CoV-2-specific host response

**DOI:** 10.55730/1300-0152.2673

**Published:** 2023-12-15

**Authors:** Rana SALİHOĞLU, Fatih SARAÇOĞLU, Mustafa SİBAİ, Talip ZENGİN, Başak ABAK MASUD, Onur KARASOY, Tuğba SÜZEK

**Affiliations:** 1 Department of Bioinformatics, University of Würzburg, Würzburg, Germany; 2 Department of Bioinformatics, Graduate School of Science and Engineering, Muğla Sıtkı Koçman University, Muğla, Turkiye; 3 Department of Computer Engineering, Faculty of Engineering, Muğla Sıtkı Koçman University, Muğla, Turkiye; 4 Josep Carreras Leukaemia Research Institute (IJC), Badalona, Spain; 5 Department of Molecular Biology and Genetics, Faculty of Science, Muğla Sıtkı Koçman University, Muğla, Turkiye

**Keywords:** Middle East respiratory syndrome coronavirus, severe acute respiratory syndrome-related coronavirus, SARS-CoV-2, web portal, principal component analysis, epithelial dysfunction

## Abstract

**Background/aim:**

Understanding the mechanism of host transcriptomic response to infection by the SARS-CoV-2 virus is crucial, especially for patients suffering from long-term effects of COVID-19, such as long COVID or pericarditis inflammation, potentially linked to side effects of the SARS-CoV-2 spike proteins. We conducted comprehensive transcriptome and enrichment analyses on lung and peripheral blood mononuclear cells (PBMCs) infected with SARS-CoV-2, as well as on SARS-CoV and MERS-CoV, to uncover shared pathways and elucidate their common disease progression and viral replication mechanisms.

**Materials and methods:**

We developed CompCorona, the first interactive online tool for visualizing gene response variance among the family Coronaviridae through 2D and 3D principal component analysis (PCA) and exploring systems biology variance using pathway plots. We also made preprocessed datasets of lungs and PBMCs infected by SARS-CoV-2, SARS-CoV, and MERS-CoV publicly available through CompCorona.

**Results:**

One remarkable finding from the lung and PBMC datasets for infections by SARS-CoV-2, but not infections by other coronaviruses (CoVs), was the significant downregulation of the angiogenin (*ANG*) and vascular endothelial growth factor A (*VEGFA*) genes, both directly involved in epithelial and vascular endothelial cell dysfunction. Suppression of the TNF signaling pathway was also observed in cells infected by SARS-CoV-2, along with simultaneous activation of complement and coagulation cascades and pertussis pathways. The ribosome pathway was found to be universally suppressed across all three viruses. The CompCorona online tool enabled the comparative analysis of 9 preprocessed host transcriptome datasets of cells infected by CoVs, revealing the specific host response differences in cases of SARS-CoV-2 infection. This included identifying markers of epithelial dysfunction via interactive 2D and 3D PCA, Venn diagrams, and pathway plots.

**Conclusion:**

Our findings suggest that infection by SARS-CoV-2 might induce pulmonary epithelial dysfunction, a phenomenon not observed in cells infected by other CoVs. The publicly available CompCorona tool, along with the preprocessed datasets of cells infected by various CoVs, constitutes a valuable resource for further research into CoV-associated syndromes.

## Graphical Abstract

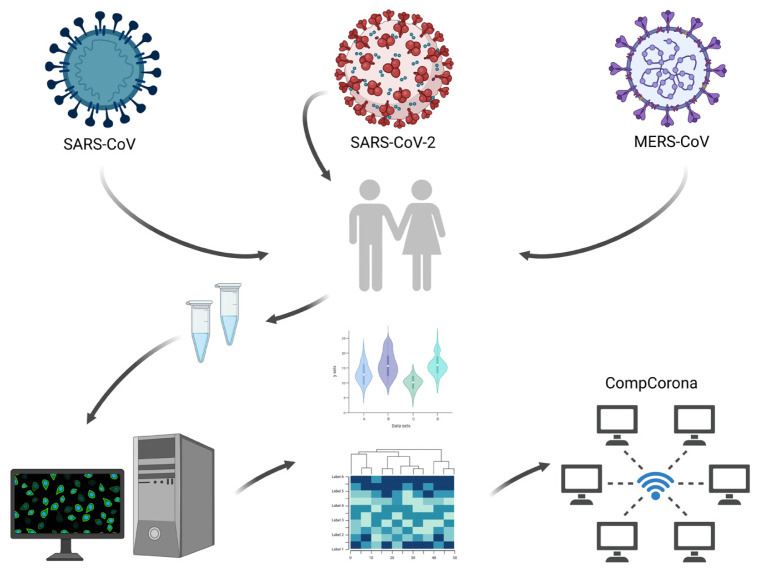

## 1. Introduction

The COVID-19 disease, stemming from severe acute respiratory syndrome coronavirus 2 (SARS-CoV-2), primarily targets the respiratory system and triggers severe immune and inflammatory responses, similar to patterns witnessed during outbreaks caused by the Middle East and severe acute respiratory syndrome coronaviruses (MERS-CoV and SARS-CoV, respectively). Unlike the seasonal endemic human coronaviruses (HCoVs) that typically cause mild respiratory responses, the three HCoVs (SARS-CoV, MERS-CoV, and SARS-CoV-2) have extended their impact beyond borders, causing widespread health crises in many nations.

Despite a noticeable decrease in the number of cases and fatalities from COVID-19 relative to the early part of 2022, largely due to a substantial rise in vaccine administrations (~ 13 billion doses; WHO, 2023), the threat of infection by SARS-CoV-2 and its variants (Kupferschmidt and Wadman, 2021), such as Delta and Omicron, persists. To understand the unique host responses to SARS-CoV-2 infection, especially given the continuously mutating variants, comparative studies have been conducted on the transcriptomes of cells infected by these three types of HCoVs (Jha et al., 2021; Krishnamoorthy et al., 2021). The differential gene expression (DEG) analyses in these studies offer insight into the molecular basis of severe lung damage and the comparatively higher frequency of cardiovascular complications associated with SARS-CoV-2 infection, as denoted by changes in several genes (e.g., *TNF*, *IL32*, *CXCL1-3*, *FOXO1*, and *TFPI2*), compared to the seasonal endemic HCoVs (Jha et al., 2021).

Moreover, shared pathways among the three HCoVs have been identified through transcriptome and enrichment analyses (KEGG and GO), suggesting various implications (e.g., neurological impact, mitochondrial dysfunction, and anosmia and olfactory dysfunction) and potential for drug repurposing (e.g., deferoxamine, verapamil, and colchicine) in addressing SARS CoV-2 infection (Krishnamoorthy et al., 2021). However, there is still a need for additional comparative studies of host responses to HCoV infections to accelerate the development of treatments for severely affected patients. In line with this, numerous investigations, including the creation of recent COVID-19 databases (e.g., COVID-19db: Zhang et al., 2022) have been undertaken.

In this study, we analyzed the transcriptome profiles of host cells infected by SARS-CoV, MERS-CoV, and SARS-CoV-2 to understand the specific host responses to SARS-CoV-2 infection. Our analysis of DEGs suggests a potential link between SARS-CoV-2 infection and various other diseases. To facilitate information sharing, we created a webpage presenting our comparative analysis of the DEGs in cells infected by SARS-CoV, MERS-CoV, and lung and peripheral blood mononuclear cell (PBMC) datasets for SARS-CoV-2 infection. Our platform also allows users to upload their own data for comparison with our datasets using Venn diagrams, and it provides interactive visualization of pertinent KEGG pathways as well as the capability to plot the principal component analysis (PCA) results of all datasets through a user-friendly web interface.

## 2. Materials and methods

### 2.1. Gene Expression Omnibus datasets for RNA sequencing analysis

Transcriptome data for cells infected by SARS-CoV-2, MERS-CoV, and SARS-CoV were acquired from the NCBI-GEO (National Center for Biotechnology Information Gene Expression Omnibus) database. The datasets included GSE147507 (representing cells infected by SARS-CoV-2), GSE139516 (representing cells infected by MERS-CoV), and GSE56192 (representing cells infected by SARS-CoV), along with additional datasets such as CRR119890 obtained from the Genome Sequence Archive (GSA). The specific sample names used in this study are given in Supplementary Table S1. All datasets were processed following the approach outlined by Griffith et al. (2015). Further details on the methodology applied, including specifics of the data processing and analysis, are provided in the Supplementary Material and Table S1.

### 2.2. RNA-Seq datasets for differential gene expression (DGE) analysis

**Figure 1:**
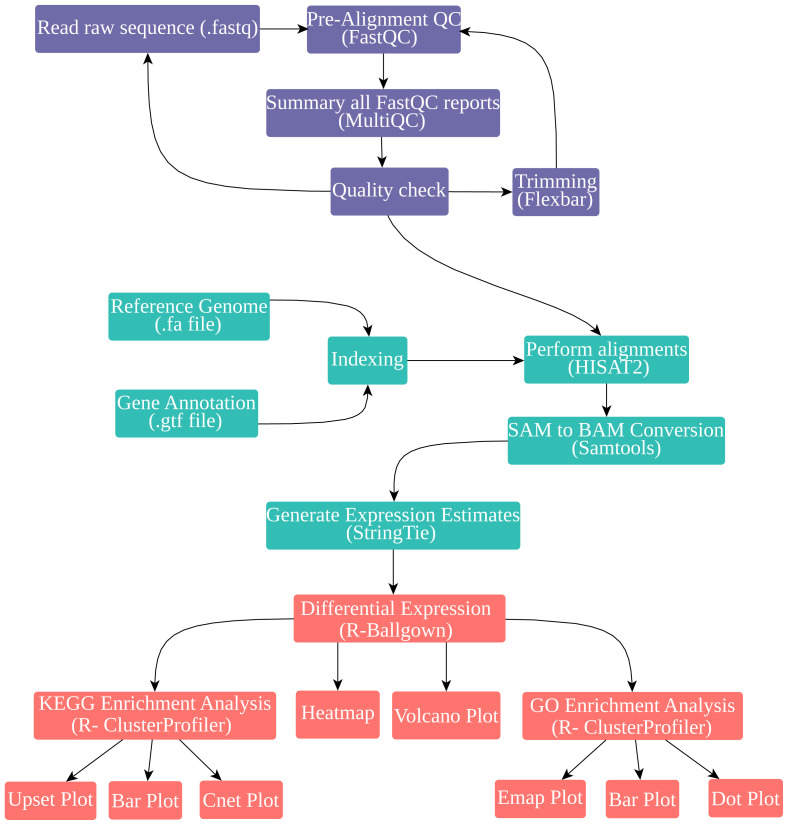
Workflow of RNA-seq, differential expression gene (DEG), Gene Ontology (GO), and Kyoto Encyclopedia of Genes and Genomes (KEGG) enrichment analysis. The figure illustrates the stepwise process employed in the study, encompassing raw RNA-seq data acquisition, quality control, preprocessing, alignment to a reference genome, identification of DEGs, and subsequent GO and KEGG enrichment analyses.

The raw data, comprising transcriptome sequences from cells infected by SARS-CoV-2, MERS-CoV, and SARS-CoV, were subjected to quality control using FastQC, complemented by several additional QC (quality control) measures. For the trimming of sequences, FLEXBAR software (v.3.4.0) was employed, as described by Dodt et al. (2012) (Figure 1). The Tuxedo pipeline (Hisat2-StringTie-Ballgown) was implemented in our analysis. This pipeline maps the reads from our datasets to the GRCh38/hg38 human genome reference using the GRCh38.99.gtf gene annotations. This approach ensured that the output files contained only human gene identifiers essential for analyzing the host cell response to viral infection. HISAT2 (v.2.1.0; Kim et al., 2015) and StringTie (v.1.3.4) tools were utilized for gene reconstructions and expression estimation. The subsequent statistical analysis of these expression estimations was conducted using the Ballgown package (v.2.20.0) within the R software environment (v.4.0.3).

### 2.3. Gene Ontology (GO) and Kyoto Encyclopedia of Genes and Genome (KEGG) enrichment analysis of DEGs

Transcriptomic analysis was performed on cells that had been infected by each of the three HCoVs: SARS-CoV-2, MERS-CoV, and SARS-CoV. For functional analysis, we utilized the clusterProfiler (version 4.2.1) and PRO-Simat tools (Wu et al., 2021; Salihoglu et al., 2023). The enrichGO and enrichKEGG functions within clusterProfiler were employed to identify enriched GO terms and KEGG pathways, respectively. Visualization of these enriched pathways was accomplished using the pathview package in the R package (Luo and Brouwer, 2013). Additionally, gene set enrichment analysis (GSEA) was performed using the clusterProfiler in the R package. For GSEA, we set the analysis with 10,000 permutations and established gene set size parameters, setting the minimum at 3 and the maximum at 600. We also applied a p-value cut-off of 0.05, which allowed for the identification of statistically significant pathways in the context of the host responses to each of the three HCoV infections.

### 2.4. Protein networks based on hub gene analysis

DEG interactions in cells infected by SARS-CoV-2, MERS-CoV, and SARS-CoV were visualized using data sourced from the STRING database, with further analysis facilitated by Cytoscape software. Within Cytoscape, the ClusterViz application and the MCODE plugin were employed to examine modules within the protein–protein interaction (PPI) networks. To identify hub genes in these PPI networks, the cytoHubba plug-in was utilized, applying the maximal clique centrality (MCC) method. This approach allowed for a detailed exploration of key genes and their interactions within the networks formed in response to infection by each of the three HCoVs.

### 2.5. Creating a web platform

An interactive web application was developed to provide user access to gene sets related to cells infected by SARS-CoV-2, MERS-CoV, and SARS-CoV. This platform allows users to upload their gene sets and visualize the data interactively. Built using the React JavaScript library, the application is designed for ease of use and efficient data handling. For the retrieval of KEGG pathway information, we utilized the BioConductor package in R, with the data being stored in a MySQL database for robust data management. The platform’s database connectivity is facilitated through the Flask framework, ensuring seamless data integration and retrieval. Additionally, the Highchart.js library is employed within the platform to enable detailed and dynamic visualization, enhancing the user’s experience in exploring and analyzing the gene sets and pathways pertinent to these CoVs.

## 3. Results

**Figure 2:**
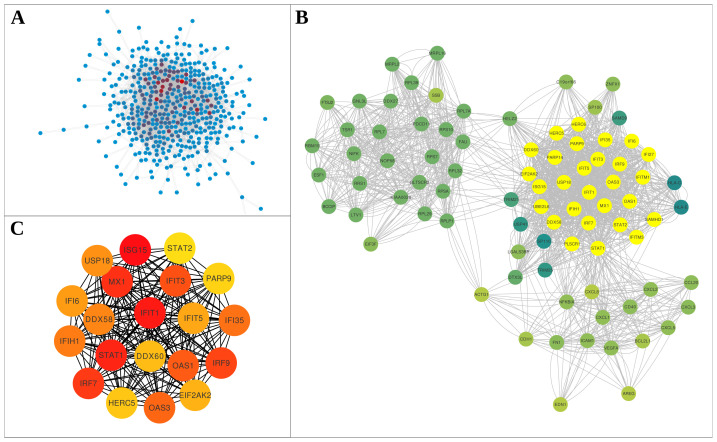
Lung SARS-CoV-2 protein–protein interactions (PPIs). **A)** Visualization of 2172 PPIs derived from StringDB, associated with 615 differentially expressed genes, using Cytoscape. **B)** Exploration of PPI network modules using the ClusterViz app with Molecular Complex Detection (MCODE), a Cytoscape plug-in. An MCODE score greater than 2 was set as the cut-off criterion, with default parameters including a degree cut-off of 2, node score cut-off of 0.2, K-core of 3, and max depth of 100. **C)** Identification of hub genes using cytoHubba plug-in within the PPI network module. The maximal clique centrality method was employed, revealing 20 hub genes. Coloring in the figure corresponds to the rank score, with the highest score represented in dark red.

We examined gene regulation during infections by SARS-CoV-2, MERS-CoV, and SARS-CoV, focusing on analyzing the transcriptome differences in human PBMCs and lung tissues infected by SARS-CoV-2 (Figure 2A). Using the MCC and MCODE methods, we identified 20 key genes specifically in the context of SARS-CoV-2 infection. These genes include *ISG15*, *IFIT1*, and *STAT1*, among others. Details of these genes and their roles in the response to SARS-CoV-2 infection are presented in Figures 2B and 2C and in Supplementary Table S2.

### 3.1. Changes in transcriptional features and functionalities of virus–host interactions in lung SARS-CoV-2 infection

In the dataset of lung tissue cells infected by SARS-CoV-2 (GSE147507), we identified 615 DEGs meeting our specific criteria (p-value < 0.05 and |log^2^(expression fold change)| ≥ 0) (Supplementary Table S3A). Among these, 335 genes were upregulated, including notable ones like *ISG15* and *MX1*, while 280 were downregulated, such as *ANG* and *NIPA1*. These genes are implicated in immune responses to viruses and other crucial biological functions.

Through GO and KEGG enrichment analysis, applying a specific threshold, we focused on the upregulated genes. The GO enrichment analysis highlighted 150 terms, divided into biological processes (115 terms), cellular components (8 terms), and molecular functions (27 terms), which collectively detail diverse virus-related responses (Supplementary Table S3B).

The downregulated genes in the lung tissue dataset for SARS-CoV-2 infection were associated with functions such as ribosome structure and cytosolic ribosome participation.

For the host response to SARS-CoV-2 infection, the upregulated DEGs were mainly enriched in pathways related to inflammation, such as the type I interferon signaling pathway. Additional enriched pathways included those related to IL-17, TNF, and NF-κB signaling pathways, among others related to viral infections.

In total, we identified 24 pathways impacted by SARS-CoV-2 in lung tissue cells. Nineteen of these pathways were activated, including pathways like legionellosis and chemokine signaling, while five, including those related to ribosomes and lysosomes, were suppressed (Supplementary Table S3C).

In comparing lung tissue infections among the different viruses, we found that 345 genes were unique to SARS-CoV-2 infection. Genes such as *IRF7* and *FXN* were highly expressed, whereas other like *GNB3* and *SURF4* were among the downregulated (Supplementary Table S3D).

### 3.2. Comparing gene expression analysis of blood and lungs for SARS-CoV-2

**Figure 3:**
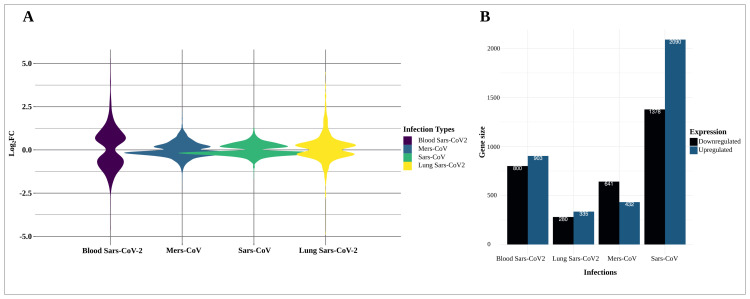
**A)** Violin plot illustrating the fold change (fc) values of differentially expressed genes obtained from SARS-CoV-2, MERS, and SARS infections. **B)** Bar plot depicting the numbers of downregulated and upregulated differentially expressed genes in SARS-CoV-2, MERS, and SARS infections.

**Figure 4:**
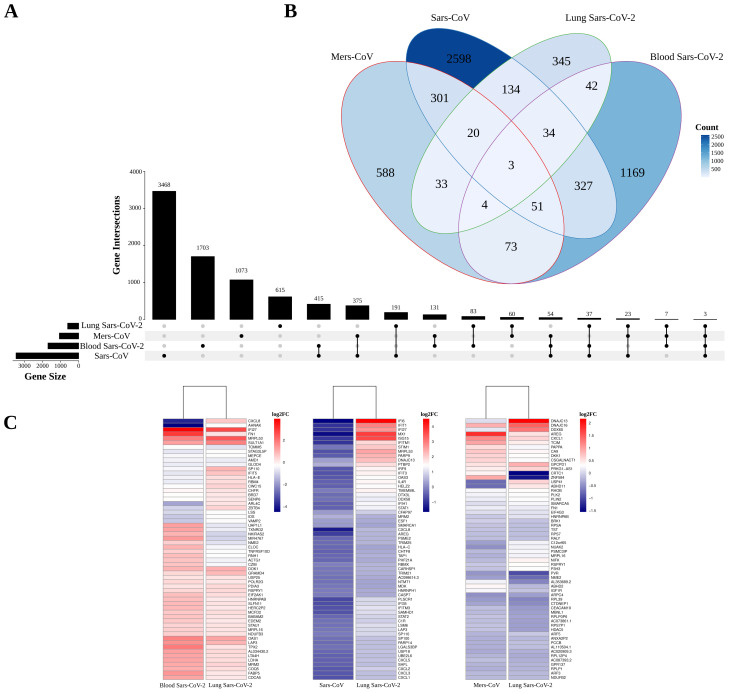
**A)** Upset plot: Visual representation of gene set intersections in SARS-CoV-2, SARS-CoV, and MERS-CoV infections, offering a comprehensive view of shared and unique genes. **B)** Venn diagram: Illustration of common and distinct genes among SARS-CoV-2, SARS-CoV, and MERS-CoV infections, highlighting shared and unique genetic components. **C)** Heatmaps: Detailed gene expression patterns within the intersections of SARS-CoV-2, SARS-CoV, and MERS-CoV infections. Red hues denote high log^2^FC values (upregulation), while blue indicates low values (downregulation).

In our comparative analysis of the PBMC dataset based on healthy individuals and patients infected by SARS-CoV-2, we identified 1703 DEGs meeting our criteria (p-value < 0.05 and |log^2^(expression fold change)| ≥ 0). Specifically, 903 genes were found to be upregulated and 800 genes were downregulated in the blood of COVID-19 patients (Figures 3A and 3B and Supplementary Table S4A). Furthermore, when comparing the transcriptome profiles between the PBMCs and lung tissues of SARS-CoV-2 patients, we found 83 DEGs that were shared between these two sample types (Figures 4A and 4B). Commonly downregulated genes in both PBMCs and lung tissues included *AHNAK*, *VAMP2*, and *CXCL8* (Figure 4C). Notably, genes such as *IFI27* and *OAS1*, which are involved in defense responses and apoptotic signaling, were upregulated in both PBMC and lung samples of COVID-19 patients. Additionally, *LAP3* and *SULT1A1* showed specific activities, while *FN1* was linked to several biological processes, including wound healing and host defense.

### 3.3. Inflammatory mediators in SARS-CoV-2

In the context of SARS-CoV-2 infection, we observed the upregulation of several chemokines and cytokines, such as *CXCL1* and *CXCL8*, which play crucial roles in inflammation and hematopoiesis. *IL-18*, a member of the IL-1 family, also exhibited increased expression, especially in activated monocytes and inflammation sites. Analysis of PBMC samples from patients with SARS-CoV-2 infection revealed the regulation of various immunoglobulin transcripts, leukocyte receptors, and genes crucial for immune response, including *RAB13* and *IFI27*. Markers indicative of inflammation were prominently evident. However, some genes were downregulated during SARS-CoV infection, including those for inflammatory cytokines like *CXCL8*, and genes involved in immune responses such as *AHNAK*. Notably, among these, the decreased expression of *DEFA1B* and *DEFA3*, genes associated with innate immunity, was observed.

### 3.4. Comparing the GO and KEGG enrichment analysis results of SARS-CoV-2 infections of PBMCs and lungs

To understand the pathogenesis of SARS-CoV-2 in lung and PBMC samples, DEGs were analyzed using GO terms. In both types of samples from SARS-CoV-2 patients, upregulated genes were predominantly associated with interferon responses, immune activity, and cytokine mediation. Conversely, downregulated genes were associated with cellular components like lysosomal lumens and inner organelle membranes. Notably, the function of natural killer cells, crucial for viral control, was found to be downregulated in PBMC samples from patients with SARS-CoV-2 infection.

From the blood samples of these patients, GO analysis identified 346 terms, subdivided into molecular functions (30 terms), biological processes (184 terms), and cellular components (132 terms) (Supplementary Table S4B). Significant upregulated genes correlated with mitochondrial activities and cellular respiration, while downregulated genes were connected to processes like histone modification and mRNA processing.

KEGG pathway analysis of the blood samples revealed that downregulated genes were associated with pathways like natural killer cell activity and RNA degradation, all with p-values of <0.001. Conversely, upregulated genes were linked to processes such as oxidative phosphorylation and diseases such as Alzheimer’s.

In summary, the gene expression pathways affected by SARS-CoV-2 infection displayed variability between lung and PBMC samples. In lung tissues, immune response pathways were predominant, whereas PBMC samples showed prominent gene modifications. Genes like *IRF7* and *PPP2R2A* emerged as potential therapeutic targets.

KEGG enrichment for metabolic alterations in patients with SARS-CoV-2 infection also revealed differences between lung and PBMC samples: lung samples showed downregulated genes affecting lysosome and ribosome pathways, while upregulated genes were tied to pathways like hepatitis C and NF-κB, with an observed increase in NF-κB activation in lung infections.

### 3.5. Transcriptome analysis of other viral infections and GO/KEGG enrichment results

#### 3.5.1. SARS-CoV

From the dataset of cells infected by SARS-CoV (GSE56192), a total of 3468 DEGs were identified (p-value < 0.05 and |log^2^(expression fold change)| ≥ 0). Of those, 2090 genes were downregulated and 1378 were upregulated (Supplementary Table S4C). Key genes in this dataset included downregulated genes such as *IFI6*, *MX1*, and *IFIT1*, as well as upregulated genes like *ACAD10*, *DMXL1*, and *MIR22*. The upregulated genes were predominantly involved in processes like cell division and nucleocytoplasmic transport. Additionally, KEGG enrichment analysis highlighted several pathways, including those related to COVID-19, PI3K-Akt, and MAPK signaling, with an enrichment of immune-related pathways observed. A total of 60 enriched pathways were identified, with 16 being activated and 44 suppressed. Among these, immune system-related pathways like TNF signaling were found to be suppressed, while others, such as those involved in the cell cycle, were upregulated.

#### 3.5.2. MERS-CoV

From the dataset of cells infected by MERS-CoV (GSE56192), we identified 1073 DEGs that met our criteria (p-value < 0.05 and |log^2^(expression fold change)| ≥ 0). Among these, 641 genes were downregulated and 432 were upregulated (Supplementary Table S4D). Notable upregulated genes included *ATF3*, *ZBTB38*, and *AREG*, while significant downregulated genes were *HCG20*, *TOMM6*, and *IFT22*. The upregulated DEGs were predominantly associated with processes like tissue morphogenesis and the steroid biosynthesis KEGG pathway. KEGG analysis also showed pathways such as those for COVID-19 and ribosomes. In total, 32 enriched pathways were identified, with 26 being activated and 6 suppressed. Activated pathways included those related to ribosomes and COVID-19, while suppressed pathways involved JAK-STAT and PI3K-Akt signaling.

In comparing the datasets of cells infected by SARS-CoV-2, SARS-CoV, and MERS-CoV, shared genes like *RPLP0P6* and *CXCL1* were identified (Supplementary Table S4E). Specifically, 60 DEGs were common between MERS-CoV and SARS-CoV-2, with notable genes including *AREG* and *CXCL1*. Additionally, 134 unique DEGs were observed between SARS-CoV and SARS-CoV-2, with genes like *STIM1* and *IFI6* linked to apoptosis and host-virus defense mechanisms (Supplementary Table S4F).

### 3.6. CompCorona: A new webpage for the analysis and visualization of CoV datasets

**Figure 5:**
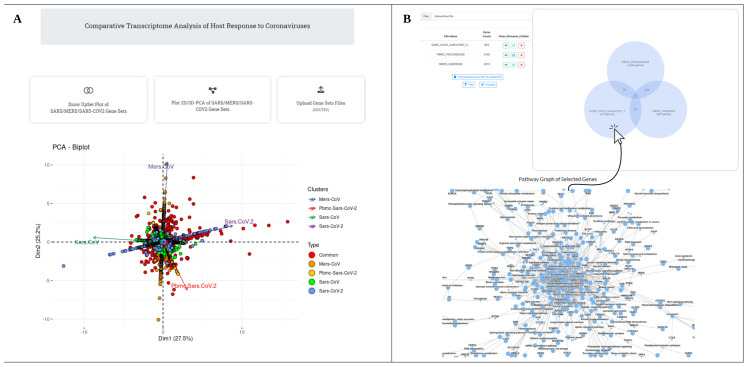
CompCorona web interface overview. **A)** The CompCorona web structure presents users with a comprehensive interface featuring a Venn diagram, 2D and 3D principal component analysis (PCA), and an interactive section for uploading differentially expressed gene (DEG) data. PCA results are illustrated as an example. PCA reveals significant variance in gene expression patterns, with *Dim1* and *Dim2* capturing 27.5% and 25.2% of the total variability, respectively. **B)** A Venn diagram is presented in a clickable format, showcasing a comparative analysis involving SARS-CoV-2, MERS-CoV, and blood-based SARS-CoV-2. Additionally, a pathway analysis result for SARS-CoV-2 DEG data is displayed, demonstrating the versatility of the platform.

CompCorona is an online platform (available at http://compcorona.mu.edu.tr) developed specifically for the comparative analysis of CoV-related datasets, including those for MERS-CoV, SARS-CoV, and SARS-CoV-2 (Figure 5). This platform is designed to display DEG data for these three CoVs, providing tools such as Venn diagram visualizations to identify common and unique DEGs across the datasets. Additionally, CompCorona facilitates the display of pathway analysis results. A key feature of the platform is its capability to offer interactive 2D and 3D PCA plots. Moreover, it allows users to upload their own DEG data for comparison and enrichment analysis, thereby enhancing its utility for researchers studying CoV infections.

## 4. Discussion

### 4.1. Common pathways between SARS-CoV-2 and other HcoVs

In our GSEA utilizing KEGG pathway enrichment, we identified 12 pathways shared between infections by SARS-CoV and SARS-CoV-2 (Supplementary Table S5A). A notable observation was the suppression of the ribosome pathway across all three CoVs, including MERS-CoV (Zhang et al., 2022). Distinct contrasts in pathway activation were noted between SARS-CoV and SARS-CoV-2, particularly in the focal adhesion and coronavirus disease pathways. In the context of COVID-19 treatment, it was found that certain therapies might increase risks for patients with Kaposi sarcoma-associated herpes virus (Chen et al., 2021). Additionally, suppression of the TNF signaling pathway was specifically observed in PBMC samples infected by SARS-CoV-2.

Shared pathways between SARS-CoV and MERS-CoV infections include those linked to Parkinson’s and osteoclast differentiation. The activation of the NOD-like receptor (NLR) signaling pathway has been observed in SARS-CoV-2 infections, and its potential connection to multiple sclerosis has been suggested (Qiu et al., 2022). Coinfections involving the Epstein–Barr virus and SARS-CoV-2 have been associated with increased fever and inflammation symptoms (Chen et al., 2021). Furthermore, reactivation of HSV-1 has been observed in severe cases of SARS-CoV-2 pneumonia (Franceschini et al., 2021). SARS-CoV-2 infections affect pathways related to immune and inflammatory responses, including the IL-17 signaling pathway. An imbalance in IL-17 can contribute to chronic inflammation and other disorders (Park et al., 2018). Targeting IL-17 may offer a potential therapeutic avenue to reduce the severity of diseases like acute respiratory distress syndrome (ARDS) in cases of SARS-CoV-2 infection (Pacha et al., 2020).

### 4.2. Down- or upregulated genes in SARS-CoV-2 and their possible implications

Transcriptomic analysis following SARS-CoV-2 infection identified a range of both downregulated and upregulated genes. The Table provides a comprehensive summary of various genes and their implications. Among the downregulated genes, *GNB3* is associated with hypertension (Chaudhary et al., 2015) and *SENP2* with newborn health issues (Nan et al., 2022). *YTHDC2*, critical in m6A function, is linked to male fertility issues following SARS-CoV-2 infections (Kamel et al., 2021). *METTL21A* plays a role in protein modification pathways, while *ANG* and *VEGFA* are essential for vascular endothelial cell function, with implications for conditions like ALS and tumor angiogenesis (Cantuti Castelvetri et al., 2020). *SURF4* affects insulin secretion, and reductions in *NIPA1* are tied to neurodegenerative diseases (Rainier et al., 2003).

On the other hand, upregulated genes include *HERC6*, involved in inflammatory responses (Paparisto et al., 2018), and *EXOG*, which is important for mitochondrial DNA repair. *FAM228B* has been linked to mental health issues (Benedetti et al., 2021), *ZNF566* to cardiovascular diseases, and *SPIRE2* to cardiovascular diseases and epilepsy (Table). Additionally, *POLR2J3*, *MMP17*, *MED17*, and *GATAD2A* are associated with various conditions ranging from infertility to neuroinflammation. *RTEL1* is linked to pulmonary fibrosis (Jenkins, 2020), *TRIM34* plays a role in restricting HIV-1 infection, *PPP2R2A* is a potential therapeutic target, and *IRF7* shows high expression in certain COVID-19 patients, underlining the diverse implications of these genes in the context of SARS-CoV-2 infection.

### 4.3. Exploring drug repurposing candidates for SARS-CoV-2

A comprehensive understanding of the pathological mechanisms of SARS-CoV-2 infection is crucial for identifying effective therapeutic interventions. In our study, we focused on elucidating the intricate pathways associated with SARS-CoV-2 by analyzing unique genes associated with the virus (Table S3D). This analysis revealed a range of molecular targets, highlighting the diverse molecular landscape the virus exploits during infection.

A crucial aspect of our investigation was the exploration of potential drug repurposing candidates based on connectivity map (CMap) analysis (Supplementary Table S5B). The drugs we identified exhibit distinct mechanisms of action, targeting key proteins associated with SARS-CoV-2 pathology. Notable among these are benzthiazide, a carbonic anhydrase inhibitor, and carvedilol (as discussed by Zhou et al., 2020; Zhang et al., 2023), an adrenergic receptor antagonist. Benzthiazide may disrupt viral pathogenesis by affecting enzymes like CA2, CA1, CA12, CA4, CA9, and the transporter SLC12A3. Carvedilol exhibits a broad spectrum of action on ADRB and ADRA receptors and affects proteins including CYP2C19, GJA1, and VEGFA.

We also highlight the potential of celecoxib (Liao, 2023) and ibuprofen as repurposing candidates. Their roles in inhibiting cyclooxygenase and modulating NF-κB pathways could mitigate the inflammatory responses associated with SARS-CoV-2 infection. Another promising candidate is zonisamide, a sodium channel and T-type calcium channel blocker, which may interfere with viral entry and replication.

While these preliminary findings highlight the potential of drug repurposing for treating SARS-CoV-2 infection, caution is necessary. Further in vitro and in vivo studies are required to confirm the efficacy and safety of these drugs specifically for COVID-19. Considerations such as pharmacokinetics, dosing regimens, and potential side effects must be thoroughly explored. Our study lays the groundwork for future research into repurposing existing drugs for SARS-CoV-2 treatment, offering new possibilities for therapeutic interventions in this ongoing global health crisis.

## 5. Conclusions

Our analysis of CoVs causing pandemics has revealed the following:

- Shared pathways between SARS-CoV and SARS-CoV-2 indicate common mechanisms in disease progression. Notably, the ribosome pathway was suppressed across MERS-CoV, SARS-CoV, and SARS-CoV-2 infections. Kaposi sarcoma-associated herpes virus reactivation could increase virus-associated cancer risks in patients treated for COVID-19.
- The suppression of the TNF signaling pathway in SARS-CoV-2 infections affects the immune response. The complement and coagulation cascades, along with pertussis pathways, show a unified immune defense response. Activation of the NLR signaling pathway in SARS-CoV-2 infections may have implications for the development of multiple sclerosis.
- The IL-17 signaling pathway, associated with ARDS, may increase cytokine expression in COVID-19 patients, presenting an opportunity for therapeutic intervention.
- Downregulation of genes such as *GNB3*, *SENP2*, and *YTHDC2* highlights potential links to hypertension, early mortality, and male fertility issues. Downregulation of *ANG*, *VEGFA*, *SURF4*, and *NIPA1* suggests impacts on neurodegenerative diseases and metabolic disorders.
- Overexpression of genes like *HERC6*, *EXOG*, and *FAM228B* offers potential therapeutic targets and insights into the broader impacts of the virus. The expression of *ZNF566* and *SPIRE2* might be related to cardiovascular diseases and epilepsy, respectively.
- Upregulation of genes such as *POLR2J3*, *MMP17*, and *MED17* provides insights into SARS-CoV-2 pathogenesis, suggesting potential links to conditions like infertility, cancers, and neuroinflammation. Our data indicate that SARS-CoV-2 might induce pulmonary epithelial senescence and fibrosis.

We have developed CompCorona, a web portal for visualizing transcriptome datasets from CoV-infected hosts. Our goal is to continually enhance CompCorona and validate SARS-CoV-2-specific mutations through in vitro experiments.

Our research offers a basis for improved therapeutic strategies against long COVID and vaccination-related myocardial damage. We encourage in vitro validation by researchers worldwide to refine disease prevention and treatment approaches, strengthening the global pandemic response.

## Code availability

https://github.com/fatihsaracoglu/compcorona/tree/master/src

https://github.com/salihoglu/CompCorona

## Supplementary Material











### 1. Materials and methods

#### 1.1. Gene Expression Omnibus datasets for RNA sequencing analysis

We sourced transcriptome data for human cells infected by SARS-CoV-2, MERS-CoV, and SARS-CoV from the National Center for Biotechnology Information Gene Expression Omnibus (NCBI-GEO) database, available at https://www.ncbi.nlm.nih.gov/geo/. Our selected datasets included GSE147507, representing postmortem lung tissue and the A549 lung cell line for SARS-CoV-2; GSE139516, correlating to lung adenocarcinoma (Calu-3) for reanalysis; and GSE56192 for both MERS-CoV and SARS-CoV (in the context of MRC5 cells).

Furthermore, accessions CRR119890, CRR125445, CRR125446, CRR119891, CRR119892, and CRR119893 were procured from the Genome Sequence Archive (GSA) at https://bigd.big.ac.cn/gsa, which contained data for SARS-CoV-2 peripheral blood mononuclear cells (PBMCs). Upon collection, these RNA sequencing datasets were subjected to rigorous analysis following the pipeline prescribed by Griffith et al. (2015).

#### 1.2. RNA-Seq datasets for differential gene expression (DGE) analysis

Quality control (QC) of the raw data was conducted utilizing FastQC (v0.11.9) and multiple additional QC measures. To enhance the precision of the sequencing results, the low-quality reads and adapters were trimmed using FLEXBAR software (v.3.4.0). This software’s approach to sequence label trimming is based on exact overlap sequence alignment, which contributes to the overall accuracy of the process (Dodt et al., 2012).

The reference genome selected for indexing in our RNA-Seq analysis was GRCh38/hg38. This is a comprehensive assembly of the human genome, first published in December 2013. To map next-generation sequencing reads to a population of human genomes and a single reference genome, we employed HISAT2 (v.2.1.0), a highly efficient and accurate alignment program (Kim et al., 2015). SAMtools (v1.9) comprises a software package and library designed for parsing and manipulating alignments stored in the SAM/BAM format. Subsequent to this, the SAM/BAM files generated by HISAT2 were analyzed using the StringTie tool (v.1.3.4), yielding precise gene reconstructions and estimations of expression levels. The data derived from this step were then aligned to human reference genome GRCh38 with the help of HISAT2 (Figure 1). Finally, for the statistical analysis of our assembled transcriptomes, we used the Ballgown package (v.2.20.0) in R (v.4.0.3). This enabled us to analyze differential gene expression (with a threshold set at p-value < 0.05 and |log^2^(expression fold change)| ≥ 0), visualize transcript structures, and match assembled transcripts to the given annotation.

#### 1.3. Gene Ontology (GO) and Kyoto Encyclopedia of Genes and Genome (KEGG) enrichment analysis of DEGs

We carried out a transcriptomic analysis of human cells infected with SARS-CoV-2, MERS-CoV, and SARS-CoV. This served to evaluate the host gene response in the face of these infections. The functional analysis and assessment of the impact of differential human gene expression across these three similar infection types were executed using the clusterProfiler package (v.4.2.1) and the PRO-Simat web tool (Wu et al., 2021; Salihoglu et al., 2023). For GO and KEGG pathway enrichment analyses, we utilized the enrichGO and enrichKEGG functions within clusterProfiler, respectively, setting a p-value threshold of less than 0.05. Subsequent visualization of the results was facilitated by the pathview R package (v.1.41.0; Luo and Brouwer, 2013). From this analytical process, we identified the most significant pathways for each type of CoV. Comparative tables and graphs were then produced to better illustrate our findings.

In order to perform gene set enrichment analysis (GSEA), we made use of the clusterProfiler package in R. This was used to annotate a list of gene names along with their corresponding log fold change values, with the genes then sorted based on these values. For the GSEA, we set the number of permutations to 10,000 and established the minimum and maximum gene set sizes as 3 and 600, respectively. A p-value cut-off was also defined at 0.05, allowing for the identification of significant pathways. The outcome of the GSEA enabled the discernment of activated and suppressed pathways. This determination was based on the normalized enrichment score attributed to each pathway.

#### 1.4. Protein networks based on hub gene analysis

The interactions among DEGs were compiled from the STRING database (http://string.embl.de/) and visualized using Cytoscape (v.3.9.1). We leveraged the NetworkAnalyzer plug-in to ascertain the characteristics of a small-world network. This was achieved by calculating key parameters, such as the distribution of the network node degree, the shortest path, the average aggregation coefficient, and the proximity to the center.

To explore protein–protein interaction (PPI) network modules, we applied the ClusterViz app with Molecular Complex Detection (MCODE), a Cytoscape plug-in. An MCODE score of >2 was established as the cut-off criterion alongside the default parameters (degree cut-off = 2, node score cut-off = 0.2, K-core = 3, max depth = 100). Finally, the cytoHubba plug-in in Cytoscape was employed to identify hub genes within the PPI network module, utilizing the maximal clique centrality (MCC) method.

#### 1.5. Creating a web platform

A new web application was devised to furnish users with interactive access to gene sets. Through this interface, users can upload their gene sets in either CSV or TSV formats and visualize their own and preanalyzed datasets via interactive Venn diagrams and PCA plots. The React JavaScript library enhances the interface’s interactivity. Importantly, uploaded files are streamed without being stored server-side and only the user receives and processes the file name and content.

In presenting the KEGG pathway information, an initial step involves gathering the pathways of all *Homo sapiens* (hsa) genes using the BioConductor package in R. The amassed data are subsequently transferred to a MySQL database. A REST service is established using the Python-based Flask framework, facilitating database connectivity. Data procured from client requests directed to this service, with gene lists and p-value parameters supplied, are transformed into a network graph by the Highchart.js library. Users can interactively access gene or pathway details by clicking on the diagram, which directs them to the corresponding NCBI information pages.[Table t1-tjb-47-06-393]

References

DodtM
RoehrJT
AhmedR
DieterichC

2012
FLEXBAR-Flexible barcode and adapter processing for next-generation sequencing platforms
Biology
1
3
895
905
10.3390/biology1030895
24832523
PMC4009805

GriffithM
WalkerJR
SpiesNC
AinscoughBJ
GriffithOL

2015
Informatics for RNA sequencing: a web resource for analysis on the cloud
PLoS Computational Biology
11
8
e1004393
10.1371/journal.pcbi.1004393
26248053
PMC4527835

KimD
LangmeadB
SalzbergSL

2015
HISAT: A fast spliced aligner with low memory requirements
Nature Methods
12
4
357
360
10.1038/nmeth.3317
25751142
PMC4655817

LuoW
BrouwerC

2013
Pathview: An R/Bioconductor package for pathway-based data integration and visualization
Bioinformatics
29
14
1830
1831
10.1093/bioinformatics/btt285
23740750
PMC3702256

SalihogluR
SrivastavaM
LiangC
SchillingK
SzalayA


2023
PRO-Simat: Protein network simulation and design tool
Computational and Structural Biotechnology Journal
21
2767
2779
10.1016/j.csbj.2023.04.023
37181657
PMC10172639

WuT
HuE
XuS
ChenM
GuoP


2021
clusterProfiler 4.0: A universal enrichment tool for interpreting omics data
Innovation
2
3
100141
10.1016/j.xinn.2021.100141
34557778
PMC8454663

## Figures and Tables

**Table t1-tjb-47-06-393:** Selected downregulated and upregulated genes in SARS-CoV-2 and their potential implications for health disorders.

Expression	Gene	Function/association	Potential implications	References
Downregulated genes	*GNB3*	Affects blood pressure responses to ACE inhibitors; linked to hypertension	Hypertension	Chaudhary et al., 2015; Angeli et al., 2022
	*SENP2*	Connected to early mortality in mice, mitochondrial dysfunction, and placental development	Newborn health issues	Nan et al., 2022
	*YTHDC2*	Key in m6A function in SARS-CoV-2; deficiency related to spermatogenesis and lung cancer	Male fertility issues following infection	Kamel et al., 2021; Hsu et al., 2017
	*METTL21A*	Linked to protein-lysine N-methyltransferase activity	Protein modification pathways	Nishizawa et al., 2014
	*ANG, VEGFA*	Vital for vascular endothelial cell function	ALS and tumor angiogenesis	Cantuti-Castelvetri et al., 2020
	*SURF4*	Reduced presence may influence insulin secretion	Insulin resistance	Saegusa et al., 2022
	*NIPA1*	Neurodegeneration	Neurodegenerative diseases	Rainier et al., 2003
Upregulated genes	*HERC6*	Known for antiviral properties: overexpression correlates with inflammation in patients	Inflammatory responses	Paparisto et al., 2018
	*EXOG*	Mitochondrial DNA repair	Mitochondrial function in treatment	Van Houten et al., 2016
	*FAM228B*	Related to brain function and depression	Mental health issues	Benedetti et al., 2021
	*ZNF566*	Role in cardiac regeneration correlated with cardioembolic stroke and cardiomyopathy	Cardiovascular diseases	Ekkert et al., 2022
	*SPIRE2*	Related to cardiovascular diseases and brain tissues	Cardiovascular diseases, epilepsy	Hao et al., 2022
	*POLR2J3*	Significance in the context of reproduction issues	Infertility and miscarriages	Taylor et al., 2017
	*MMP17*	Shedding process of ACE2	Several cancers, cardiovascular diseases, and inflammation	Xiao et al., 2022
	*MED17*	Role in miRNA expression modulation during viroid pathogenesis	Not investigated	Nozawa et al., 2017
	*GATAD2A*	Related to impaired autophagy and increased H1N1 replication	Neuroinflammation, metabolic and thyroid issues	Ma et al., 2018
	*RTEL1*	Related to pulmonary fibrosis	Lung damage	Jenkins, 2020
	*TRIM34*	Known to restrict HIV-1 infection	Viral infection	Wang et al., 2022
	*PPP2R2A*	Regulatory subunit promoting Th1 and Th17 differentiation towards PP2A	Potential therapeutic target for conditions associated with Th1 and Th17 cell proliferation	Pan et al., 2021
	*IRF7*	Highly expressed in patients	Immune responses	Scagnolari et al., 2021

## Data Availability

In the Supplementary Material file http://compcorona.mu.edu.tr Tutorial: https://youtu.be/JE4-SXZHoPQ

## References

[b38-tjb-47-06-393] Dodt M Roehr JT Ahmed R Dieterich C 2012 FLEXBAR-Flexible barcode and adapter processing for next-generation sequencing platforms Biology 1 3 895 905 10.3390/biology1030895 24832523 PMC4009805

[b39-tjb-47-06-393] Griffith M Walker JR Spies NC Ainscough BJ Griffith OL 2015 Informatics for RNA sequencing: a web resource for analysis on the cloud PLoS Computational Biology 11 8 e1004393 10.1371/journal.pcbi.1004393 26248053 PMC4527835

[b40-tjb-47-06-393] Kim D Langmead B Salzberg SL 2015 HISAT: A fast spliced aligner with low memory requirements Nature Methods 12 4 357 360 10.1038/nmeth.3317 25751142 PMC4655817

[b41-tjb-47-06-393] Luo W Brouwer C 2013 Pathview: An R/Bioconductor package for pathway-based data integration and visualization Bioinformatics 29 14 1830 1831 10.1093/bioinformatics/btt285 23740750 PMC3702256

[b42-tjb-47-06-393] Salihoglu R Srivastava M Liang C Schilling K Szalay A 2023 PRO-Simat: Protein network simulation and design tool Computational and Structural Biotechnology Journal 21 2767 2779 10.1016/j.csbj.2023.04.023 37181657 PMC10172639

[b43-tjb-47-06-393] Wu T Hu E Xu S Chen M Guo P 2021 clusterProfiler 4.0: A universal enrichment tool for interpreting omics data Innovation 2 3 100141 10.1016/j.xinn.2021.100141 34557778 PMC8454663

